# Isolated Intrinsic Ureteral Endometriosis: A Rare Presentation of Ureteral Obstruction

**DOI:** 10.7759/cureus.18919

**Published:** 2021-10-20

**Authors:** Vishal Bahall, Yasmin Hosein, Siva Konduru, Mickhaiel Barrow

**Affiliations:** 1 Obstetrics and Gynaecology, The University of the West Indies, St Augustine, TTO; 2 Obstetrics and Gynaecology, San Fernando General Hospital, San Fernando, TTO; 3 Obstetrics and Gynaecology, Port of Spain General Hospital, Port of Spain, TTO; 4 Radiology, Scarborough General Hospital, Scarborough, TTO; 5 Pathology, Port of Spain General Hospital, Port of Spain, TTO

**Keywords:** endometriosis and chronic pelvic pain, unilateral urinary obstruction, ureteral anastamosis, ureteric obstruction, intrinsic ureteral endometriosis

## Abstract

Intrinsic ureteral endometriosis is a very rare presentation of deep infiltrating endometriosis. It can lead to urinary tract obstruction and loss of renal function. Clinical suspicion and radiologic assessment can aid in preoperative diagnosis and help plan surgical treatment. Herein we report a case of a 29-year-old female who presented with left-sided pelvic and flank pain. Imaging revealed left obstructive uropathy and a left ureteral mass. She underwent laparotomy and resection of the diseased ureter with primary re-anastomosis. Histology confirmed intrinsic ureteral endometriosis. There was subsequently complete resolution on follow-up imaging, with no permanent loss of renal function.

## Introduction

Endometriosis is defined as the presence of endometrial glands and stroma outside the uterus [1]. This endometrial-like tissue becomes implanted outside of the uterus by several proposed theories, the most common being that of retrograde menstruation where endometrial cells enter the peritoneal cavity via the fallopian tubes. Although ectopically implanted, the tissue maintains responses similar to that of the endometrium. The overall prevalence is unknown and varies widely among different groups of patients. It has been found in approximately 2%-10% of the general population, up to 32% in women undergoing laparoscopy for chronic pelvic pain and up to 50% of women with infertility [2,3]. Endometriosis can be classified as superficial peritoneal, ovarian or deep infiltrating endometriosis (DIE) [4]. DIE is a specific form of endometriosis that penetrates >5mm under the peritoneal surface and can involve the rectovaginal space, the uterosacral ligaments and organs such as the bowel and urinary tract. The bladder is the most common site of urinary tract involvement, with ureteral involvement only in approximately 1% of all endometriosis patients [5]. Intrinsic ureteral endometriosis is even rarer, occurring in only one-fifth of all cases of endometriosis of the ureter [6].

The diagnosis of ureteral endometriosis can be difficult since urinary symptoms are present in more common conditions and the typical symptoms of endometriosis may not be present. This can be misleading and often lead to a delay in diagnosis. Furthermore, ureteral endometriosis may be asymptomatic in up to 40% of cases and discovered during investigation for renal impairment [7].

We report the first case of isolated intrinsic ureteral endometriosis in the Caribbean in a young woman with no prior history of endometriosis and no typical endometriosis symptoms.

## Case presentation

A 29-year-old primi-parous East Indian presented to her gynaecologist with a two-month history of worsening left pelvic pain. The pain was non-cyclical and she experienced concomitant left flank pain. She had no history of abnormal bleeding, abnormal vaginal discharge or urinary symptoms. She had no significant past medical history. She had no previous surgery but has a family history (sister and mother) of endometriosis. Clinical examination revealed left pelvic tenderness and left adnexal tenderness only, with no other significant findings.

**Figure 1:**
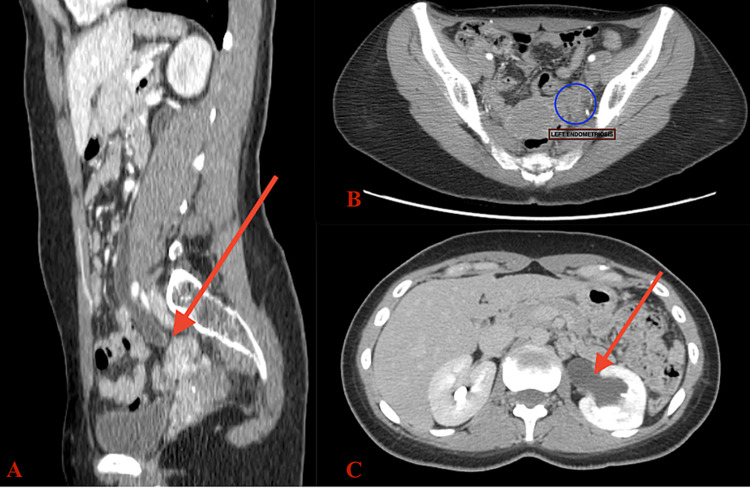
Contrast computed tomography (CT) scan showing (A) sagittal view of the abdomen with left hydroureter extending to mass (arrow), (B) axial view of left endometriotic mass (circle) and (C) axial view at the level of kidney demonstrating dilated left renal pelvis and severe left hydronephrosis.

The pelvic ultrasound scan done was unremarkable. A contrast computed tomography (CT) of the abdomen and pelvis was requested due to the suspicion of a left obstructing ureteral stone. CT scan reported left hydronephrosis and hydroureter extending to the left adnexa up to a 3.1x2.8 cm spiculated mass that was inseparable from the ureter (Figures 1A-1C). There was no flow of contrast seen in the dilated left ureter. Her blood investigations revealed normal - renal function (creatinine 0.6mg/dL, BUN - 13mg/dL), electrolytes (sodium - 138mmol/L, potassium - 4.1mmol/L, chloride - 105mmol/L) and complete blood count (haemoglobin - 13.0g/dL). Due to her age and family history of endometriosis, a presumptive diagnosis of left ureteral obstruction secondary to ureteral endometriosis was made based on the CT findings.

**Figure 2:**
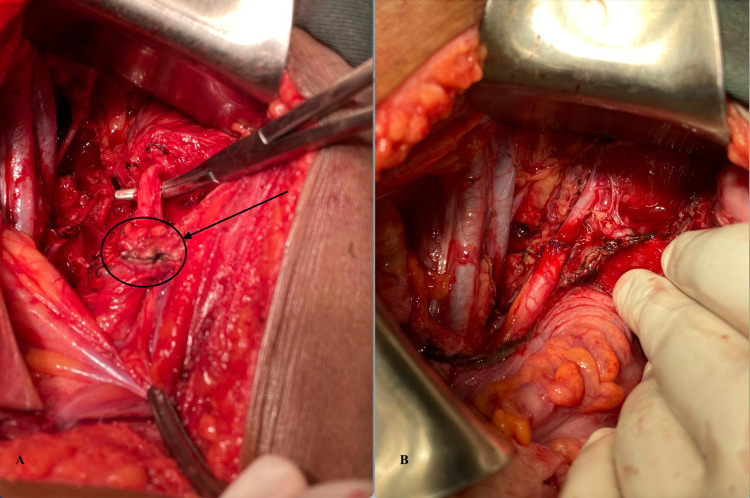
Intra-operative images showing (A) left obstructing endometriotic mass with left hydroureter and (B) ureter post-resection and primary uretero-ureteral anastomosis.

**Figure 3:**
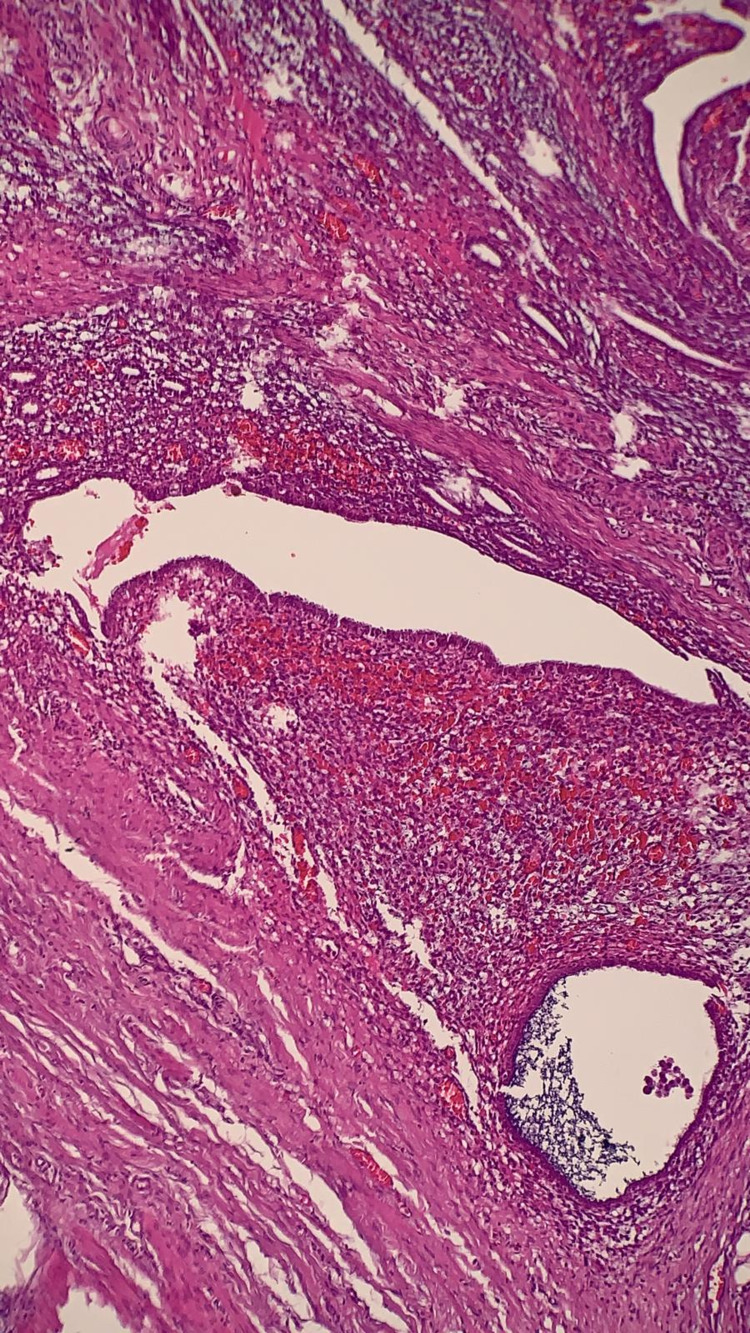
Histology confirming endometrioid glands surrounded by stroma and haemorrhage embedded within the tunica muscularis of the ureter (400x higher magnification).

The case was reviewed with the radiologist and a decision to proceed with the surgical intervention was made to relieve the complete ureteral obstruction. Intra-operatively, the uterus, ovaries and tubes showed no evidence of endometriosis. There was a lesion seen over the left sidewall, the was firm and irregular. The left retro-peritoneal space was opened revealing left hydronephrosis and an isolated lesion that was invading the ureter. There was no evidence of any other abnormal lesions or endometriosis in the pelvis or right retro-peritoneal spaces. The left ureter was then isolated up to the uterine vessels. The mass along with the diseased ureteral segment (Figure 2A) was excised (partial ureterectomy). The distal and proximal ends of the transected ureter were spatulated. A ureteral stent was inserted so that each end was in the kidney and bladder respectively. A primary uretero-ureteral anastomosis was performed using an interrupted 4-0 polyglactin suture (Figure 2B). Histopathology of the specimen showed endometrial glands surrounded by stroma and haemorrhage embedded within the muscularis layer of the ureter (Figure 3). There was no evidence of malignancy. This confirmed intrinsic ureteral endometriosis.

The ureteral stent was removed six weeks post-operatively. Renal ultrasound and a diethylene triamine penta-acetic acid (DTPA) renogram were done which showed good renal function bilaterally and no obstruction. The patient was started on Dienogest 2mg daily after histology confirmed endometriosis. Four months later, the patient is still well, asymptomatic and there is no indication of recurrence.

## Discussion

Extragenital endometriosis is rarely involved in the urinary tract but when it does, the ureter is the second commonest site and is involved in 10% of cases [8]. Histologically, ureteral endometriosis can be classified as intrinsic or extrinsic [6]. Intrinsic ureteral endometriosis is defined by the presence of endometrial tissue in the lamina propria or tunica muscularis of the ureter. Ureteral endometriosis is considered extrinsic when DIE lesions involve the peri-ureteral tissue and result in ureteral obstruction. The pathogenesis of ureteral endometriosis is still unclear but the most common theory is that of retrograde menstruation [6]. This theory also supports the predilection of endometriosis for the lower third and left ureter [7]. This was observed in our patient where she had involvement of the left ureter. The sigmoid colon on the left side decreases fluid movement in the left hemi-pelvis and under the influence of gravity, the endometrium regurgitated by the fallopian tubes are encouraged to adhere and grow with a propensity for the left distal ureter [9]. The increased incidence of endometriosis in first-degree relatives of women with the disease also suggests a hereditary component as was seen in our patient where both her mother and her sister had endometriosis.

The symptoms of endometriosis are dependent on the site and extent of lesions. Dysmenorrhea, chronic pelvic pain, deep dyspareunia, cyclical intestinal complaints, fatigue and infertility continue to be the leading symptoms of endometriosis [10]. The symptoms of urinary tract endometriosis are nonspecific and can be misleading. Soriano et al. (2011) found that 95.5% of their patients with ureteral endometriosis had dysmenorrhea, 60% had dyspareunia while only 15.9% of patients experienced urinary tract symptoms [11]. Rare presentations reported in the literature include unexplained hypertension [12] and anuria [13]. In the case presented above, the only symptom was chronic flank and pelvic pain. A tentative diagnosis of a ureteral stone was made in this case based on the patient’s symptoms.

Pre-operative diagnosis of intrinsic ureteral endometriosis can be difficult. In one study, ureteral endometriosis was presumptively diagnosed before surgery in only 40% of patients [14]. A transvaginal ultrasound was done on this patient but it was normal. In other cases, transvaginal ultrasound can reveal the presence of endometriomas and therefore is still indicated. A renal ultrasound can identify urinary tract obstruction. CT scan is the most commonly used imaging modality in low-resource settings such as ours. A CT scan with contrast of the abdomen and pelvis in this patient was instrumental in providing a preoperative diagnosis. It was able to determine the extent, size and location of the mass and identify urinary tract obstruction. This along with the patient’s age and family history strongly suggested the mass to be endometriosis. Another important differential to consider is malignancy. However, in a young, healthy patient, this is unlikely. This case proves that good radiological support is necessary to obtain an early diagnosis.

Ureteroscopy is also used to aid in the diagnosis, particularly of intrinsic endometriosis [15]. Apart from the identification of endometriotic lesions, ureteroscopy allows for biopsy, histological confirmation and ablation [16]. It may also help to measure the distance between the lower endometriotic margins and ureteral orifices which are of significance for the choice of surgical approach [17]. Intravenous pyelography (IVP) and retrograde pyelography have also been traditionally used but magnetic resonance imaging (MRI) has since replaced these [9]. MRI remains the investigation of choice in assessing disease extension and deciding on the surgical approach [18]. Sillou et al. (2015) in a retrospective study compared MRI to surgery for detecting intrinsic endometriosis. MRI had a sensitivity of 91% but only a specificity of 59% as MRI overestimated intrinsic disease (21 intrinsic lesions diagnosed by MRI but only 10 confirmed on the histology) [19].

Due to its rarity, there are no clear guidelines for managing ureteral endometriosis. Patients should be individually managed and the aim is the relieve symptoms, preserve renal function and prevent relapse.

Medical management in the form of hormonal contraceptives, progestogens and gonadotropin-releasing hormone agonists have a role in early-stage disease for temporary relief. The response to hormonal therapy is incomplete owing to the desmoplastic reaction within the layers of the uterus [20]. The relapse rate is also high after the cessation of hormonal treatment [9]. Medical management only is contraindicated in severe hydronephrosis secondary to ureteral endometriosis because of the risk of progression and loss of renal function [9].

Surgical management with ureteral resection and reconstruction is indicated in cases of intrinsic endometriosis especially in the setting of severe hydronephrosis. The two basic techniques include ureteral-ureteral anastomosis and ureteroneocystostomy. The first technique should be performed if the ureteral disease is limited and the distal ureter can be preserved [13]. However, there might be a higher risk of recurrence in this technique [21]. Ureteroneocystostomy is the operation of choice in cases of extended disease with ureteral disease close to the vesicoureteral junction, usually in cases where the distal stump is less than 1 cm [22,23]. In this case, we opted to perform the first technique as there was enough distal ureter to provide anastomosis without tension, as is the aim of either technique. Nephrectomy unfortunately still occurs in up to 47% of cases and is necessary in cases of deteriorated renal function or in cases where the diagnosis is still unclear and the lesions mimic a carcinoma [24]. Seraccholi et al. (2015) in their analysis of a large series of women affected by DIE found that up to 90% of women with ureteral endometriosis had endometriosis in other sites [14]. This is vastly contrary to the index case where there was absolutely no endometriosis seen at any other sites intraoperatively. Intrinsic ureteral endometriosis is a silent cause of renal failure [14]. Greater awareness of this condition is needed for early diagnosis and management. Physicians should suspect ureteral endometriosis in women of reproductive age with obstructive uropathy and a ureteral mass. Early recognition and surgical intervention followed by long-term medical management of endometriosis lead to favourable outcomes with no loss in renal function.

## Conclusions

Intrinsic ureteral endometriosis poses a diagnostic challenge due to its vague symptomatology and rare incidence. Its symptoms can range from classic symptoms of endometriosis (most common) to urinary tract symptoms (less common). The index case presented with urinary tract symptoms thus making the diagnosis more elusive. A greater awareness in literature is needed to educate clinicians on the variable presentations of endometriosis. This will allow for prompt diagnosis and treatment so that permanent loss of renal function can be avoided. In this case, good radiologic support assisted the pre-operative diagnosis since the symptoms were inconsistent with endometriosis. Long-term medical management after surgical removal of the mass and ureteric reconstruction is essential to decrease recurrence in these women.
